# Association of endomyocardial fibrosis and minor myocarditis sequelae with intracardiac thrombus and Ebstein like valvulopathy in a patient with Behçet disease: a case report

**DOI:** 10.1093/ehjcr/ytad631

**Published:** 2023-12-20

**Authors:** Hélène Ceruti, Philippe van de Borne, Daniela-Corina Mirica

**Affiliations:** Department of Cardiology, Hopital Universitaire de Bruxelles (HUB), Route de Lennik, Hopital Erasme, Route de Lennik 808, 1070 Anderlecht, Bruxelles, Belgium; Department of Cardiology, Hopital Universitaire de Bruxelles (HUB), Route de Lennik, Hopital Erasme, Route de Lennik 808, 1070 Anderlecht, Bruxelles, Belgium; Department of Cardiology, Hopital Delta, Boulevard du Triomphe Hopital Delta, Boulevard du Triomphe 201, 1160 Auderghem, Bruxelles, Belgium

**Keywords:** Behçet disease, Intracardiac thrombus, Endomyocardial fibrosis, Ebstein disease, Myocarditis, Tricuspid insufficiency, Case report

## Abstract

**Background:**

Cardiac complications occur in 1–6% of cases of Behçet disease (BD) with intracardiac thrombus being the most frequent complication. Endomyocardial fibrosis, less common and occasionally associated with intracardiac thrombus, is reported in <20 case reports of BD, among which, three cases are described to mimic Ebstein disease based on echocardiography. We present the first case in the literature of a 34-year-old man with BD diagnosed with multiple cardiovascular complications, highlighting the challenging diagnosis and treatment of this pathology, especially regarding anticoagulation therapy.

**Case summary:**

A 34-year-old man, diagnosed with BD, presented to the Emergency Room with haemoptysis. Computed tomography study of the thorax diagnosed pulmonary arterial aneurysm with multiple arterial thrombi, associated with multiple intracardiac thrombi in the right ventricle and atrium. The echocardiography confirmed the presence of voluminous thrombi in the right ventricle and atrium and showed hypertrabeculation of the right ventricle and a high insertion of the posterior leaflet of the tricuspid valve inducing a moderate tricuspid insufficiency compatible with an Ebstein disease. The cardiac MRI later revealed right ventricular fibrosis consistent with endomyocardial fibrosis and sequelae of myocarditis, also described as BD rare cardiac manifestations. The patient had a favourable outcome under anticoagulant treatment and immunosuppressive drugs.

**Discussion:**

The association of multiple cardiovascular complications can occur in a single patient with BD. The endomyocardial fibrosis in the right heart chambers acting as a substrate for thrombus formation and subsequent pulmonary embolism; fibrosis extending to the tricuspid valve inducing an Ebstein-like morphology.

**Learning points**

- Screening for cardiovascular complications in Behçet disease (BD), it is an important element as their presence leads to poor prognosis.
- Endomyocardial fibrosis in the right ventricle in BD may lead in early stages to fibrous tumours and thrombus formation, and its extension to the tricuspid valve may induce an Ebstein-like morphology; extensive fibrosis leads to right heart failure.
- Early detection of fibrosis by echocardiography or more precisely by MRI with early immunosuppressive treatment may improve prognosis.

## Introduction

Behçet disease (BD) is a multi-systematic vasculitis characterized by lesions of veins and arteries of all sizes with a predominant damage to oral, genital and ocular tissue.^1^ Cardiac involvement is rare—between 1% and 6% of cases in clinical series and presents with a broad spectrum of manifestations including pericarditis, myocarditis, valvulopathy, endomyocardial fibrosis (EMF), and intracardiac thrombosis, usually related with a poor prognosis. We present the case of a 34-year-old man suffering from BD who was diagnosed with multiple cardiovascular complications.

## Summary figure

**Figure:**
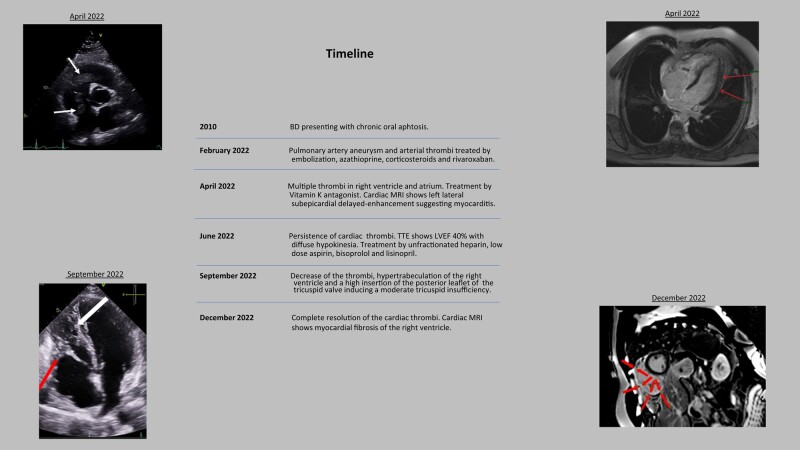


## Case presentation

A 34-year-old North African man, with a medical history of BD, presented to the Emergency Department in February 2022 with a two-week history of haemoptysis and dry cough. He had no signs of active BD and was not on any medical treatment. Blood pressure at admission was 110/70 mmHg; heart rate was 100 b.p.m.; and oxygen saturation was 99%.

Upon physical exam, a tricuspid regurgitant murmur was revealed. There were no signs of right-sided cardiac failure. NT-proBNP was 737 ng/L (<125 ng/L). C-reactive protein was 88 mg/L (<5 mg/L). A thoracic contrast enhanced computed tomography (CT) scan showed a right posterior basal segmental artery aneurysm measuring 12 mm of diameter, associated with multiple arterial thrombi in the segmental and sub-segmental arteries of the inferior lobe. The COVID-19 PCR assay was negative. The patient was initially treated by embolization with a favourable clinical response. He was discharged from the hospital with azathioprine 2 mg/kg/day, oral corticosteroids (1 mg/kg), and rivaroxaban 20 mg.

**Figure 1:**
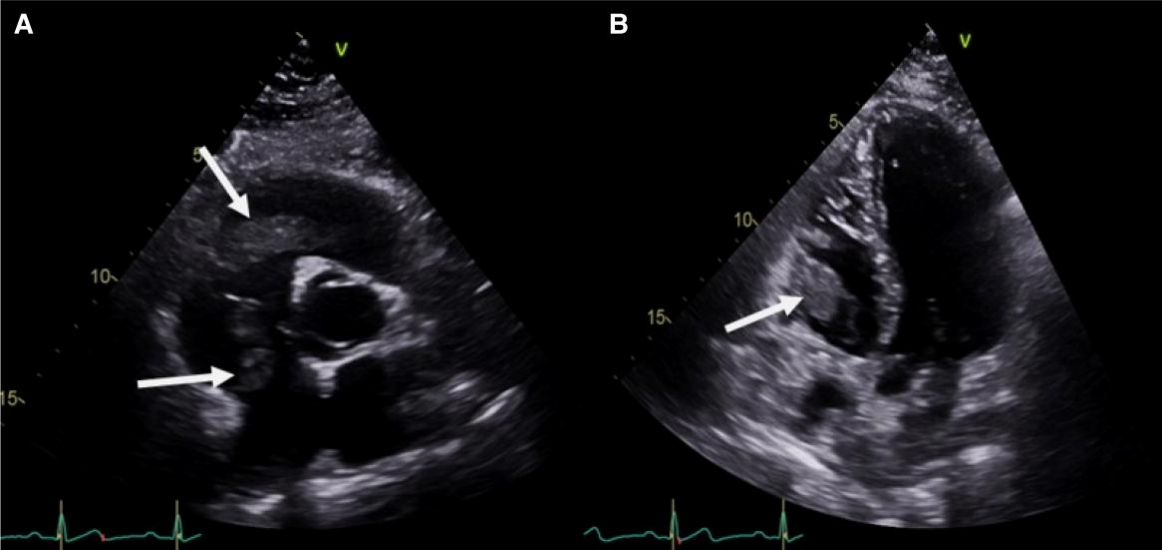
Two-dimensional transthoracic echocardiography reveals multiple thrombi in the right cavities (*A*)—parasternal short axis view showing one thrombus in the right atrium close to the septal leaflet of the tricuspid valve and one thrombus in the right ventricle attached to the lateral region of the tricuspid annulus (arrow)—(*B*) apical four-chamber view showing the thrombus at the level of the free wall of the right ventricle (arrow).

Two months later, he was readmitted to the pneumology department for recurrent haemoptysis. A new thoracic CT scan showed a left segmental pulmonary embolism, resolution of the aneurysms, and hypodense material in the right ventricle compatible with a thrombus. A transthoracic echocardiogram (TTE) confirmed the diagnosis of multiple endocavitary thrombi: the largest one of 41 × 16 mm located at the level of the free wall of the right ventricular attached to the lateral region of the tricuspid annulus, one at the level of the right ventricular apex, and one of 18 × 14 mm in the right atrium close to the septal leaflet of the tricuspid valve (*Figure 1* and Supplementary material online, *Video S1*). Left and right ventricular systolic functions were normal, and there was a moderate tricuspid insufficiency.

**Figure 2:**
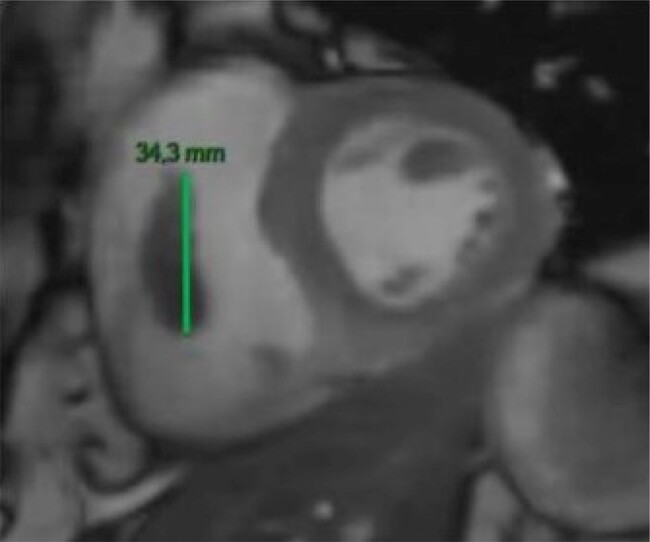
Cardiac magnetic resonance image showing right intraventricular thrombus.

A cardiac magnetic resonance imaging (MRI) was performed 7 days later and confirmed the presence of two large thrombi—one of 16 × 13 mm in the right atrium and one of 34 × 11 mm attached to the lateral trabeculations of the right ventricle (*Figure 2* and Supplementary material online, *Videos S2* and *S3*). Left ventricular ejection fraction (LVEF) was of 45% with diffuse hypokinesia and a discrete left lateral subepicardial delayed-hyper-enhancement suggesting a sequela of myocarditis. Treatment with a vitamin K antagonist (VKA)—acenocoumarol—was initiated and the patient was dismissed.

The patient was readmitted 2 months later to the department of cardiology for New York Heart Association (NYHA) functional class 3 dyspnoea. A TTE was repeated showing persistence of right cardiac thrombi in spite of well-conducted VKA treatment. Left ventricular ejection fraction was 40% with diffuse hypokinesia. Given the persistence of cardiac thrombi, VKA was replaced by intravenous unfractionated heparin and aspirin 80 mg was added for its antithrombotic action. Bisoprolol 2.5 mg and lisinopril 2.5 mg twice a day were added to the therapy. UFH was administered for 2 weeks, daily monitored by the APPT. Acenocoumarol was reintroduced, and the patient was discharged on VKA.

**Figure 3:**
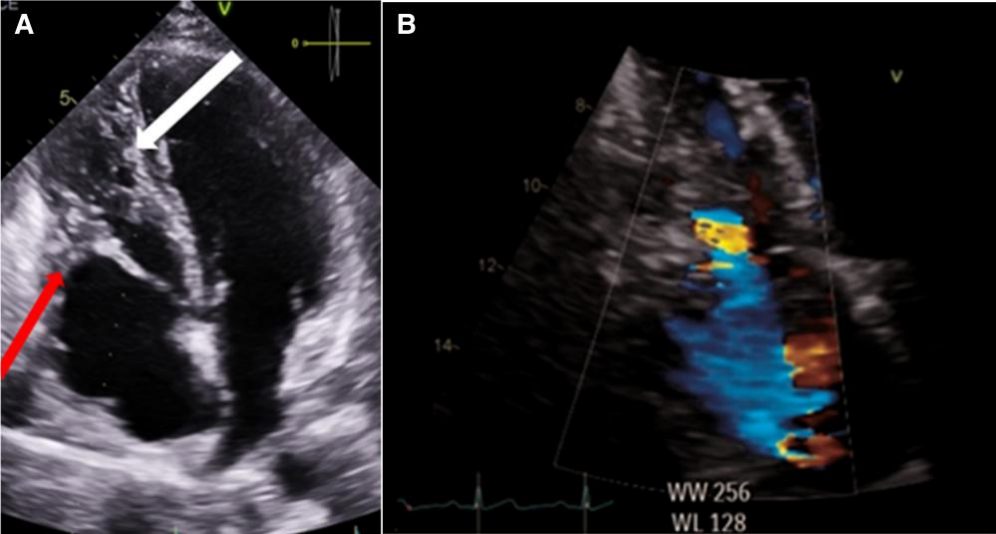
Apical four-chamber view in transthoracic echocardiography indicating (*A*) hypertrabeculation of the right ventricle (arrow), a high insertion of the posterior leaflet of the tricuspid valve ( arrow), a dilated right atrium—(*B*) moderate tricuspid regurgitation.

**Figure 4:**
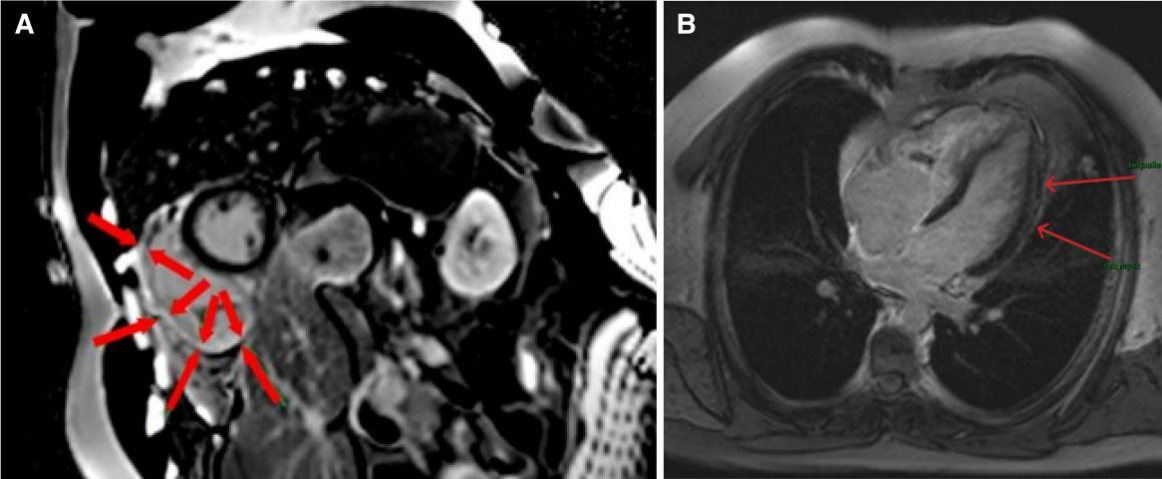
(*A*) Delayed gadolinium enhancement cardiac MRI of short axis view shows subendocardial hyper-enhancement of the right ventricle suggesting fibrosis (arrows)—(*B*) delayed gadolinium enhancement cardiac MRI shows discrete left lateral subepicardial hyper-enhancement suggesting a sequela of myocarditis.

After 3 months, the patient was asymptomatic at the cardiac ambulatory follow-up, with a TTE demonstrating an obvious decrease in the size of the two thrombi, hypertrabeculation of the right ventricle and a high insertion of the posterior leaflet of the tricuspid valve inducing a moderate tricuspid insufficiency mimicking an Ebstein disease (ED) (*Figure 3*). The LVEF had returned to normal. A new cardiac MRI showed in comparison with the previous examination: a nearly complete resolution of the cardiac thrombi, new onset of subendocardial delayed-enhancement of the right ventricle suggesting myocardial fibrosis and an improvement of the LVEF (50%) (*Figure 4*). There were no anomalies of implantation of the tricuspid valve, and there was persistence of left lateral subepicardial delayed-hyper-enhancement suggesting a sequela of myocarditis.

The patient was still treated by VKA, aspirin, azathioprine, and oral corticosteroids. Bisoprolol and lisinopril were discontinued at the patient’s request.

## Discussion

Our case report depicts the multiple cardiac involvement that can occur in patients with BD. Appropriate diagnosis and treatment of these conditions can be challenging and usually requires multimodality imaging and the association of different treatment modalities, especially concerning anticoagulant therapy.

Behçet disease is a multi-systematic inflammatory disease characterized by the following triad: genital ulcerations, oral aphthae, and ocular involvement. The disease is mainly found in young adults 20 to 40 years of age around the Mediterranean basin and in Japan, frequently associated with the human leukocyte antigen (HLA)-B51.^1^ The diagnosis is mainly clinical and is based on the International Study Group for BD criteria since the lack of pathognomonic laboratory tests. Treatment is individually targeted, based on organ involvement and on the severity of symptoms and consists of anti-inflammatory therapies and/or immunosuppressive drugs.

Cardiac involvement is rare and occurs in 1% to 6% of cases, and possibly more (up to 16%) according to a Japanese autopsy registry.^2^ Overall survival in patients with cardiac involvement is decreased.^3^ The main reported clinical presentations are pericarditis, valvular disease, myocarditis, intracardiac thrombosis, and EMF.^3–5^ Cardiovascular involvement includes venous and arterial vasculitis lesions, with a predominance for venous involvement.^6^

Intracardiac thrombus has been reported as the most frequent clinical feature, with a predominance of male cases and with the right ventricular being the first site involved; the right atrium being the second one.^7^ Pulmonary embolism can be associated in 60% of cases.^3^ There are currently no established guidelines regarding the exact therapy. Treatment of intracardiac thrombus usually consists of anticoagulation therapy such as warfarin, acetylsalicylic acid plus corticosteroids and/or other immunosuppressant drug therapy for complete resolution of the thrombus, suggesting that vascular inflammation plays a major role in thrombus formation.^3,8,9^

Myocarditis is also a common feature in BD and in our case it could explain the lower LVEF of the patient, especially given the LVEF recovery after appropriate medication. Another explanation for the left ventricle dysfunction could be Behçet cardiomyopathy. Systolic and diastolic heart failure has been described as a cardiac complication of BD. In fact, strain rates in BD are known to be decreased in comparison to control group.^6^ However, this diagnosis seems less possible given the fact that the patient was under immunosuppressive treatment at the time of the cardiac MRI.

Several cases of EMF in association with BD have also been described, frequently associated with cardiac thrombi (*Table 1*).^10,11^ In most cases, the lesions were discovered incidentally during routine TTE with a right ventricle that appeared as a bright echogenic pseudotumor, with displacement of the leaflets of the tricuspid or mitral valve and right atrial enlargement as seen in our case. Final diagnosis is usually made by biopsy or surgery but is not always necessary given the TTE and MRI typical features.^12^ Heart failure was present in most of the cases, with a majority of right-sided disease. Treatment remains controversial with regard to long-term results. Surgery is suggested in cases of EMF with heart failure and consists in surgical excision of fibrotic plaques with or without valvular intervention.^13^

**Table 1:** Main features of patients with Behçet disease and Ebstein-like valvulopathy and/or endomyocardial fibrosis

Case report	Age, sex, ethnicity	Intracardiac thrombus	Pulmonary embolism	Fibrosis of right cavities	Valvulopathy	Cardiac failure	Ebstein disease like	Myocarditis	Intracardiac thrombus treatment
Buturak *et al*.^14^	26 years, male, Turkish	Absent	Absent	Present on MRI	Mild tricuspid insufficiency	Present	Present	Absent	Absent
Vaidyanathan *et al*.^13^	14 years, male, unknown	Absent	Absent	Present during surgery	Moderate tricuspid insufficiency	Absent	Present	Absent	Absent
Ceruti *et al*.	34 years, male, Moroccan	Present	Present	Present on MRI	Moderate tricuspid insufficiency	Absent	Present	Present	1/Vit K antagonist + aspirin 2/Azathioprine and corticosteroids
Houman *et al*.^15^	29 years, male, Tunisian	Present	Present	Present on endomyocardial biopsy	Tricuspid insufficiency	Absent	Absent	Absent	1/Vit K antagonist 2/Colchicine, corticosteroids, and cyclophosphamide
Mirfezei *et al*.^12^	34 years, female, unknown	Absent	Absent	Present on biopsy	Tricuspid insufficiency	Present	Absent	Absent	Absent
Huong *et al*.^11^	34 years, male, Algerian	Absent	Absent	Present on biopsy	Tricuspid insufficiency	Present	Present	Absent	Absent
Huong *et al*.^11^	32 years, female, French	Absent	Absent	Present on biopsy	Mitral insufficiency	Absent	Absent	Absent	Absent
Huong *et al*.^11^	24 years, male, Turkish	Absent	Absent	Present on biopsy	Severe tricuspid insufficiency	Present	Absent	Absent	Absent
Huong *et al*.^11^	27 years, male, Algerian	Absent	Absent	Present on biopsy	Tricuspid insufficiency	Present	Absent	Absent	Absent

Our case report is the fourth case described in the literature to mimick Ebstein disease due to an abnormal tricuspid valve. ED is a rare congenital condition characterized by apical displacement of the insertion of the posterior and septal leaflets of the tricuspid valve responsible for tricuspid insufficiency associated with right ventricular dysfunction.^13,12^ Our case report depicts a possible case of ED based on the high insertion of the posterior leaflet of the tricuspid valve inducing a moderate tricuspid insufficiency as seen on the TTE. The diagnosis was however challenged by the cardiac MRI that showed no valve implantation defect but revealed right ventricular fibrosis consistent with EMF. Buturak *et al*. explain that EMF has a progressive nature because of chronic, relapsing inflammatory pattern of the syndrome of Behçet. This probably explains why in some patients without heart failure, lesions of EMF resolved after use of immunosuppressive agents, possibly delaying progression of disease.^3^

A thorough follow-up is needed in our patient, in order to monitor the myocardial fibrosis and the tricuspid insufficiency, due to the possible progression of fibrosis, which may be correlated to the degree of chronic inflammation caused by the disease.

## Conclusion

Multiple cardiac involvement of BD in a single patient is extremely rare and usually associated with a poor prognosis. Intracardiac thrombus is the most frequent clinical feature and is often associated with EMF, which can rarely mimic ED. Early diagnosis of EMF is made using TTE and MRI and should be treated, by immunosuppressive agents.

## Supplementary Material

ytad631_Supplementary_Data

## Data Availability

The data underlying this article are available in the article and in its online Supplementary material.

## Lead author biography

My name is Hélène Ceruti and am an actual resident in cardiology at the Universite Libre de Bruxelles. My imterests include echocardiography, structural cardiomyopathies, and preventive cardiology. I am happy to join the family of the young European cardiologists since I will be graduating at the end of the actual year.

## Notes

**Consent**: The authors confirm that written consent for the submission and publication of this case, including images, has been obtained from the patient in line with COPE guidance

**Funding:** None declared.
