# Supplementary material for: Association of endomyocardial fibrosis and minor myocarditis sequelae with intracardiac thrombus and Ebstein like valvulopathy in a patient with Behçet disease: a case report
Source: Eur Heart J Case Rep. 2023 Dec 20;8(1):ytad631. doi: 10.1093/ehjcr/ytad631 (PMC10762881; doi:10.1093/ehjcr/ytad631)
Supplement: ytad631_Supplementary_Data [file ytad631_supplementary_data.zip › Videos.docx]

**Videos**

- Video 1 : Short axis transthoracic echography showing Intracardiac thrombus in right atrium and right ventricle

- Video 2 : Cardiac MRI cine image depicting intracardiac thrombus in right atrium

- Video 3: Cardiac MRI cine image depicting intracardiac thrombus in right ventricle
