# Supplementary material for: Association of endomyocardial fibrosis and minor myocarditis sequelae with intracardiac thrombus and Ebstein like valvulopathy in a patient with Behçet disease: a case report
Source: Eur Heart J Case Rep. 2023 Dec 20;8(1):ytad631. doi: 10.1093/ehjcr/ytad631 (PMC10762881; doi:10.1093/ehjcr/ytad631)
Supplement: ytad631_Supplementary_Data [file ytad631_supplementary_data.zip › Supplementary files 12-11.docx]

**Supplementary files**

- 4 cavities transthoracic echography (zoom on the tricuspid valve) showing moderate Tricuspid valve regurgitation

- 4 cavities transthoracic echography showing resolution of thrombi, hypertrabeculation of the right ventricle and a high insertion of the posterior leaflet of the tricuspid valve inducing a moderate tricuspid insufficiency
